# (3, 2)-Fuzzy Sets and Their Applications to Topology and Optimal Choices

**DOI:** 10.1155/2021/1272266

**Published:** 2021-10-27

**Authors:** Hariwan Z. Ibrahim, Tareq M. Al-shami, O. G. Elbarbary

**Affiliations:** ^1^Department of Mathematics, Faculty of Education, University of Zakho, Zakho, Iraq; ^2^Department of Mathematics, Sana'a University, Sana'a, Yemen; ^3^Department of Mathematics, Faculty of Science, Tanta University, Tanta, Egypt

## Abstract

The purpose of this paper is to define the concept of (3, 2)-fuzzy sets and discuss their relationship with other kinds of fuzzy sets. We describe some of the basic set operations on (3, 2)-fuzzy sets. (3, 2)-Fuzzy sets can deal with more uncertain situations than Pythagorean and intuitionistic fuzzy sets because of their larger range of describing the membership grades. Furthermore, we familiarize the notion of (3, 2)-fuzzy topological space and discuss the master properties of (3, 2)-fuzzy continuous maps. Then, we introduce the concept of (3, 2)-fuzzy points and study some types of separation axioms in (3, 2)-fuzzy topological space. Moreover, we establish the idea of relation in (3, 2)-fuzzy set and present some properties. Ultimately, on the basis of academic performance, the decision-making approach of student placement is presented via the proposed (3, 2)-fuzzy relation to ascertain the suitability of colleges to applicants.

## 1. Introduction

The concept of fuzzy sets was proposed by Zadeh [1]. The theory of fuzzy sets has several applications in real-life situations, and many scholars have researched fuzzy set theory. After the introduction of the concept of fuzzy sets, several research studies were conducted on the generalizations of fuzzy sets. The integration between fuzzy sets and some uncertainty approaches such as soft sets and rough sets has been discussed in [2–4].

The idea of intuitionistic fuzzy sets suggested by Atanassov [5] is one of the extensions of fuzzy sets with better applicability. Applications of intuitionistic fuzzy sets appear in various fields, including medical diagnosis, optimization problems, and multicriteria decision making [6–8]. Yager [9] offered a new fuzzy set called a Pythagorean fuzzy set, which is the generalization of intuitionistic fuzzy sets. Fermatean fuzzy sets were introduced by Senapati and Yager [10], and they also defined basic operations over the Fermatean fuzzy sets.

The concept of fuzzy topological spaces was introduced by Chang [11]. He studied the topological concepts like continuity and compactness via fuzzy topological spaces. Then, Lowen [12] presented a new type of fuzzy topological spaces. Çoker [13] subsequently initiated a study of intuitionistic fuzzy topological spaces. Recently, Olgun et al. [14] presented the concept of Pythagorean fuzzy topological spaces and Ibrahim [15] defined the concept of Fermatean fuzzy topological spaces.

The main purpose of this paper is to introduce the concept of (3, 2)-fuzzy sets and compare them with the other types of fuzzy sets. We introduce the set of operations for the (3, 2)-fuzzy sets and explore their main features. Following the idea of Chang, we define a topological structure via (3, 2)-fuzzy sets as an extension of fuzzy topological space, intuitionistic fuzzy topological space, and Pythagorean fuzzy topological space. We discuss the main topological concepts in (3, 2)-fuzzy topological spaces such as continuity and compactness. In addition, the concept of relation to (3, 2)-fuzzy sets is investigated. Finally, an improved version of max-min-max composite relation for (3, 2)-fuzzy sets is proposed.

## 2. (3, 2)-Fuzzy Sets

In this section, we initiate the notion of (3, 2)-fuzzy sets and study their relationship with other kinds of fuzzy sets. Then, we furnish some operations to (3, 2)-fuzzy sets.

**Definition 1 .**

Let *X* be a universal set. Then, the (3, 2)-fuzzy set (briefly, (3, 2)-FS) *D* is defined by the following:

(1) $$\begin{array}{c} D=\left\{\langle r,\alpha_{D}\left(r\right),\beta_{D}\left(r\right)\rangle :\;r\in X\right\}, \end{array}$$

where *α*_*D*_(*r*) : *X*⟶[0,1] is the degree of membership and *β*_*D*_(*r*) : *X*⟶[0,1] is the degree of non-membership of *r* ∈ *X* to *D*, with the condition

(2) $$\begin{array}{c} 0\leq {\left(\alpha_{D}\left(r\right)\right)}^{3}+{\left(\beta_{D}\left(r\right)\right)}^{2}\leq 1. \end{array}$$

The degree of indeterminacy of *r* ∈ *X* to *D* is defined by

(3) $$\begin{array}{c} \pi_{D}\left(r\right)=\sqrt[{5}]{1-\left[{\left(\alpha_{D}\left(r\right)\right)}^{3}+{\left(\beta_{D}\left(r\right)\right)}^{2}\right]}. \end{array}$$

It is clear that (*α*_*D*_(*r*))^3^+(*β*_*D*_(*r*))^2^+(*π*_*D*_(*r*))^5^=1, and *π*_*D*_(*r*)=0 whenever (*α*_*D*_(*r*))^3^+(*β*_*D*_(*r*))^2^=1. In the interest of simplicity, we shall mention the symbol *D*=(*α*_*D*_, *β*_*D*_) for the (3, 2)-FS *D*={〈*r*, *α*_*D*_(*r*), *β*_*D*_(*r*)〉 :  *r* ∈ *X*}.

**Definition 2 .**

Let *X* be a universal set. Then, the intuitionistic fuzzy set (IFS) [5] (resp. Pythagorean fuzzy set (PFS) [9] and Fermatean fuzzy set (FFS) [10]) is defined by the following:

(4) $$\begin{array}{c} K=\left\{\langle r,\alpha_{K}\left(r\right),\beta_{K}\left(r\right)\rangle :\;r\in X\right\}, \end{array}$$

with the condition 0 ≤ *α*_*K*_(*r*)+*β*_*K*_(*r*) ≤ 1 (resp. 0 ≤ (*α*_*K*_(*r*))^2^+(*β*_*K*_(*r*))^2^ ≤ 1, 0 ≤ (*α*_*K*_(*r*))^3^+(*β*_*K*_(*r*))^3^ ≤ 1), where *α*_*K*_(*r*) : *X*⟶[0,1] is the degree of membership and *β*_*K*_(*r*) : *X*⟶[0,1] is the degree of non-membership of every *r* ∈ *X* to *K*.

To illustrate the importance of (3, 2)-FS to extend the grades of membership and non-membership degrees, assume that *α*_*D*_(*r*)=0.9 and *β*_*D*_(*r*)=0.5 for *X*={*r*}. We obtain 0.9+0.5=1.40 > 1 and (0.9)^2^+(0.5)^2^=1.06 > 1 which means that *D*=(0.9, 0.5) neither follows the condition of IFS nor follows the condition of PFS. On the other hand, (0.9)^3^+(0.5)^2^=0.979 < 1 which means we can apply the (3, 2)-FS to control it. That is, *D*=(0.9, 0.5) is a (3, 2)-FS.

**Theorem 1 .**

The set of (3, 2)-fuzzy membership grades is larger than the set of intuitionistic membership grades and Pythagorean membership grades.

**Proof**

It is well known that for any two numbers *r*_1_, *r*_2_ ∈ [0,1], we have

(5) $$\begin{array}{c} r_{1}^{3}\leq r_{1}^{2}\leq r_{1},\\ r_{2}^{2}\leq r_{2}. \end{array}$$

Then, we get

(6) $$\begin{array}{c} r_{1}+r_{2}\leq 1\\ \Rightarrow r_{1}^{2}+r_{2}^{2}\leq 1\\ \Rightarrow r_{1}^{3}+r_{2}^{2}\leq 1. \end{array}$$

**Figure 1:**
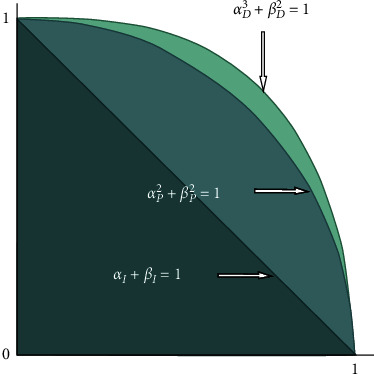
Comparison of grade space of IFSs, PFSs, and (3, 2)-FSs.

Hence, the space of (3, 2)-fuzzy membership grades is larger than the space of intuitionistic membership grades and Pythagorean membership grades. This development can be evidently recognized in Figure 1.

**Lemma 1 .**

Let *X*={*r*_*j*_ :  *j*=1,…, *k*} be a universal set and *D* be (3, 2)-FS. If *π*_*D*_(*r*_*j*_)=0, then $\left|\alpha_{D}\left(r_{j}\right)\right|=\sqrt[{3}]{\left|\left(\beta_{D}\left(r_{j}\right)-1\right)\left(\beta_{D}\left(r_{j}\right)+1\right)\right|}$.

**Proof**

Presume that *D* is (3, 2)-FS and *π*_*D*_(*r*_*j*_)=0 for *r*_*j*_ ∈ *X*; then,

(7) $$\begin{array}{c} {\left(\alpha_{D}\left(r_{j}\right)\right)}^{3}+{\left(\beta_{D}\left(r_{j}\right)\right)}^{2}=1\Rightarrow -{\left(\alpha_{D}\left(r_{j}\right)\right)}^{3}={\left(\beta_{D}\left(r_{j}\right)\right)}^{2}-1\Rightarrow -{\left(\alpha_{D}\left(r_{j}\right)\right)}^{3}=\left(\beta_{D}\left(r_{j}\right)-1\right)\left(\beta_{D}\left(r_{j}\right)+1\right)\Rightarrow \left|{\left(\alpha_{D}\left(r_{j}\right)\right)}^{3}\right|\\ =\left|\left(\beta_{D}\left(r_{j}\right)-1\right)\left(\beta_{D}\left(r_{j}\right)+1\right)\right|\Rightarrow {\left|\alpha_{D}\left(r_{j}\right)\right|}^{3}=\left|\left(\beta_{D}\left(r_{j}\right)-1\right)\left(\beta_{D}\left(r_{j}\right)+1\right)\right|\Rightarrow \left|\alpha_{D}\left(r_{j}\right)\right|=\sqrt[{3}]{\left|\left(\beta_{D}\left(r_{j}\right)-1\right)\left(\beta_{D}\left(r_{j}\right)+1\right)\right|}. \end{array}$$

**Example 1 .**

Let *D* be (3, 2)-FS and *r* ∈ *X* such that *β*_*D*_(*r*)=0.82 and *π*_*D*_(*r*)=0. Then, $\left|\alpha_{D}\left(r\right)\right|=\sqrt[{3}]{\left|\left(\beta_{D}\left(r\right)-1\right)\left(\beta_{D}\left(r\right)+1\right)\right|}=\sqrt[{3}]{\left|\left(-0.18\right)\left(1.82\right)\right|}=\sqrt[{3}]{0.3276}$.

**Definition 3 .**

Let *δ* be a positive real number (*δ* > 0). If *D*_1_=(*α*_*D*_1__, *β*_*D*_1__) and *D*_2_=(*α*_*D*_2__, *β*_*D*_2__) are two (3, 2)-FSs, then their operations are defined as follows:

1. *D*_1_∩*D*_2_=(min{*α*_*D*_1__, *α*_*D*_2__}, max{*β*_*D*_1__, *β*_*D*_2__}).
2. *D*_1_ ∪ *D*_2_=(max{*α*_*D*_1__, *α*_*D*_2__}, min{*β*_*D*_1__, *β*_*D*_2__}).
3. *D*_1_^*c*^=(*β*_*D*_1__, *α*_*D*_1__).
4. $\delta D_{1}=\left(\sqrt[{5}]{1-{\left(1-\alpha_{D_{1}}^{3}\right)}^{\delta }},\beta_{D_{1}}^{\delta }\right)$.
5. $D_{1}^{\delta }=\left(\alpha_{D_{1}}^{\delta },\sqrt[{5}]{1-{\left(1-\beta_{D_{1}}^{2}\right)}^{\delta }}\right)$.

**Remark 1 .**

We will use supremum “sup” instead of maximum “max” and infimum “inf” instead of minimum “min” if the union and the intersection are infinite.

**Example 2 .**

Assume that *D*_1_=(*α*_*D*_1__=0.9, *β*_*D*_1__=0.5) and *D*_2_=(*α*_*D*_2__=0.89, *β*_*D*_2__=0.49) are both (3, 2)-FSs. Then,

1. *D*_1_∩*D*_2_=(min{*α*_*D*_1__, *α*_*D*_2__}, max{*β*_*D*_1__, *β*_*D*_2__})=(min{0.9, 0.89}, max{0.5, 0.49})=(0.89, 0.5).
2. *D*_1_ ∪ *D*_2_=(max{*α*_*D*_1__, *α*_*D*_2__}, min{*β*_*D*_1__, *β*_*D*_2__})=(max{0.9, 0.89}, min{0.5, 0.49})=(0.9, 0.49).
3. *D*_1_^*c*^=(0.5, 0.9).
4. $\delta D_{1}=\left(\sqrt[{5}]{1-{\left(1-\alpha_{D_{1}}^{3}\right)}^{\delta }},\beta_{D_{1}}^{\delta }\right)=\left(\sqrt[{5}]{1-{\left(1-0.9^{3}\right)}^{4}},0.5^{4}\right)\approx \left(0.99892,0.06250\right)$, for *δ*=4.
5. $D_{1}^{\delta }=\left(\alpha_{D_{1}}^{\delta },\sqrt[{5}]{1-{\left(1-\beta_{D_{1}}^{2}\right)}^{\delta }}\right)=\left(0.9^{4},\sqrt[{5}]{1-{\left(1-0.5^{2}\right)}^{4}}\right)\approx \left(0.65610,0.92674\right)$, for *δ*=4.

**Theorem 2 .**

Let *L*_1_=(*α*_*L*_1__, *β*_*L*_1__) and *L*_2_=(*α*_*L*_2__, *β*_*L*_2__) be two (3, 2)-FSs; then, the following properties hold:

1. *L*_1_∩*L*_2_=*L*_2_∩*L*_1_.
2. *L*_1_ ∪ *L*_2_=*L*_2_ ∪ *L*_1_.
3. (*L*_1_∩*L*_2_) ∪ *L*_2_=*L*_2_.
4. (*L*_1_ ∪ *L*_2_)∩*L*_2_=*L*_2_.

**Proof**

From Definition 3, we can obtain

1. *L*_1_∩*L*_2_=(min{*α*_*L*_1__, *α*_*L*_2__}, max{*β*_*L*_1__, *β*_*L*_2__})=(min{*α*_*L*_2__, *α*_*L*_1__}, max{*β*_*L*_2__, *β*_*L*_1__})=*L*_2_∩*L*_1_.
2. The proof is similar to (1).
3. (*L*_1_∩*L*_2_) ∪ *L*_2_=(min{*α*_*L*_1__, *α*_*L*_2__}, max{*β*_*L*_1__, *β*_*L*_2__}) ∪ (*α*_*L*_2__, *β*_*L*_2__)=(max{min{*α*_*L*_1__, *α*_*L*_2__}, *α*_*L*_2__}, min{max{*β*_*L*_1__, *β*_*L*_2__}, *β*_*L*_2__})=(*α*_*L*_2__, *β*_*L*_2__)=*L*_2_.
4. The proof is similar to (3).

**Theorem 3 .**

Let *L*_1_=(*α*_*L*_1__, *β*_*L*_1__), *L*_2_=(*α*_*L*_2__, *β*_*L*_2__) and *L*_3_=(*α*_*L*_3__, *β*_*L*_3__) be three (3, 2)-FSs and *δ* > 0; then,

1. *L*_1_∩(*L*_2_∩*L*_3_)=(*L*_1_∩*L*_2_)∩*L*_3_.
2. *L*_1_ ∪ (*L*_2_ ∪ *L*_3_)=(*L*_1_ ∪ *L*_2_) ∪ *L*_3_.
3. *δ*(*L*_1_ ∪ *L*_2_)=*δL*_1_ ∪ *δL*_2_.
4. (*L*_1_ ∪ *L*_2_)^*δ*^=*L*_1_^*δ*^ ∪ *L*_2_^*δ*^.

**Proof**

For the three (3, 2)-FSs *L*_1_, *L*_2_, and *L*_3_ and *δ* > 0, according to Definition 3, we can obtain

(1) (8) $$\begin{array}{c} L_{1}\cap \left(L_{2}\cap L_{3}\right)=\left(\alpha_{L_{1}},\beta_{L_{1}}\right)\cap \left(\min\left\{\alpha_{L_{2}},\alpha_{L_{3}}\right\},\max\left\{\beta_{L_{2}},\beta_{L_{3}}\right\}\right)=\left(\min\left\{\alpha_{L_{1}},\min\left\{\alpha_{L_{2}},\alpha_{L_{3}}\right\}\right\},\max\left\{\beta_{L_{1}},\max\left\{\beta_{L_{2}},\beta_{L_{3}}\right\}\right\}\right)\\ =\left(\min\left\{\min\left\{\alpha_{L_{1}},\alpha_{L_{2}}\right\},\alpha_{L_{3}}\right\},\max\left\{\max\left\{\beta_{L_{1}},\beta_{L_{2}}\right\},\beta_{L_{3}}\right\}\right)\\ =\left(\min\left\{\alpha_{L_{1}},\alpha_{L_{2}}\right\},\max\left\{\beta_{L_{1}},\beta_{L_{2}}\right\}\right)\cap \left(\alpha_{L_{3}},\beta_{L_{3}}\right)=\left(L_{1}\cap L_{2}\right)\cap L_{3}. \end{array}$$
(2) The proof is similar to (1).
(3) (9) $$\begin{array}{c} \delta \left(L_{1}\cup L_{2}\right)=\delta \left(\max\left\{\alpha_{L_{1}},\alpha_{L_{2}}\right\},\min\left\{\beta_{L_{1}},\beta_{L_{2}}\right\}\right)=\left(\sqrt[{5}]{1-{\left(1-\max\left\{\alpha_{L_{1}}^{3},\alpha_{L_{2}}^{3}\right\}\right)}^{\delta }},\min\left\{\beta_{L_{1}}^{\delta },\beta_{L_{2}}^{\delta }\right\}\right),\\ \delta L_{1}\cup \delta L_{2}=\left(\sqrt[{5}]{1-{\left(1-\alpha_{L_{1}}^{3}\right)}^{\delta }},\beta_{L_{1}}^{\delta }\right)\cup \left(\sqrt[{5}]{1-{\left(1-\alpha_{L_{2}}^{3}\right)}^{\delta }},\beta_{L_{2}}^{\delta }\right)\\ =\left(\max\left\{\sqrt[{5}]{1-{\left(1-\alpha_{L_{1}}^{3}\right)}^{\delta }},\sqrt[{5}]{1-{\left(1-\alpha_{L_{2}}^{3}\right)}^{\delta }}\right\},\min\left\{\beta_{L_{1}}^{\delta },\beta_{L_{2}}^{\delta }\right\}\right)\\ =\left(\sqrt[{5}]{1-{\left(1-\max\left\{\alpha_{L_{1}}^{3},\alpha_{L_{2}}^{3}\right\}\right)}^{\delta }},\min\left\{\beta_{L_{1}}^{\delta },\beta_{L_{2}}^{\delta }\right\}\right)=\delta \left(L_{1}\cup L_{2}\right). \end{array}$$
(4) The proof is similar to (3).

In the following result, we claim that *L*^*c*^ is (3, 2)-FS for any (3, 2)-FS *L*.

**Theorem 4 .**

Let *L*_1_=(*α*_*L*_1__, *β*_*L*_1__) and *L*_2_=(*α*_*L*_2__, *β*_*L*_2__) be two (3, 2)-FSs such that *L*_1_^*c*^ and *L*_2_^*c*^ are (3, 2)-FSs. Then,

1. (*L*_1_∩*L*_2_)^*c*^=*L*_1_^*c*^ ∪ *L*_2_^*c*^.
2. (*L*_1_ ∪ *L*_2_)^*c*^=*L*_1_^*c*^∩*L*_2_^*c*^.

**Proof**

For the two (3, 2)-FSs *L*_1_ and *L*_2_, according to Definition 3, we can obtain

(1) (10) $$\begin{array}{c} {\left(L_{1}\cap L_{2}\right)}^{c}={\left(\min\left\{\alpha_{L_{1}},\alpha_{L_{2}}\right\},\max\left\{\beta_{L_{1}},\beta_{L_{2}}\right\}\right)}^{c}=\left(\max\left\{\beta_{L_{1}},\beta_{L_{2}}\right\}\right.,\\ \left.\min\left\{\alpha_{L_{1}},\alpha_{L_{2}}\right\}\right)=\left(\beta_{L_{1}},\alpha_{L_{1}}\right)\cup \left(\beta_{L_{2}},\alpha_{L_{2}}\right)=L_{1}^{c}\cup L_{2}^{c}. \end{array}$$
(2) The proof is similar to (1).

**Definition 4 .**

Let *D*_1_=(*α*_*D*_1__, *β*_*D*_1__) and *D*_2_=(*α*_*D*_2__, *β*_*D*_2__) be two (3, 2)-FSs; then,

1. *D*_1_=*D*_2_ if and only if *α*_*D*_1__=*α*_*D*_2__ and *β*_*D*_1__=*β*_*D*_2__.
2. *D*_1_ ≥ *D*_2_ if and only if *α*_*D*_1__ ≥ *α*_*D*_2__ and *β*_*D*_1__ ≤ *β*_*D*_2__.
3. *D*_2_ ⊂ *D*_1_ or *D*_1_⊃*D*_2_ if *D*_1_ ≥ *D*_2_.

**Example 3 .**

1. If *D*_1_=(0.9, 0.5) and *D*_2_=(0.9, 0.5) for *X*={*x*}, then *D*_1_=*D*_2_.
2. If *D*_1_=(0.9, 0.5) and *D*_2_=(0.81, 0.61) for *X*={*x*}, then *D*_2_ ≤ *D*_1_ and *D*_2_ ⊂ *D*_1_.

## 3. Topology with respect to (3, 2)-Fuzzy Sets

In this section, we formulate the concept of (3, 2)-fuzzy topology on the family of (3, 2)-fuzzy sets whose complements are (3, 2)-fuzzy sets and scrutinize main properties. Then, we define (3, 2)-fuzzy continuous maps and give some characterizations. Finally, we establish two types of (3, 2)-fuzzy separation axioms and reveal the relationships between them.

### 3.1. (3, 2)-Fuzzy Topology

**Definition 5 .**

Let *τ* be a family of (3, 2)-fuzzy subsets of a non-empty set *X*. If

1. 1_*X*_, 0_*X*_ ∈ *τ* where 1_*X*_=(1,0) and 0_*X*_=(0,1),
2. *D*_1_∩*D*_2_ ∈ *τ*, for any *D*_1_, *D*_2_ ∈ *τ*,
3. ∪_*i*∈*I*_*D*_*i*_ ∈ *τ*, for any {*D*_*i*_}_*i*∈*I*_ ⊂ *τ*,

then *τ* is called a (3, 2)-fuzzy topology on *X* and (*X*, *τ*) is a (3, 2)-fuzzy topological space. We call *D* an open (3, 2)-FS if it is a member of *τ* and call its complement a closed (3, 2)-FS.

**Remark 2 .**

We call *τ*={1_*X*_, 0_*X*_} the indiscreet (3, 2)-fuzzy topology on *X*. If *τ* contains all (3, 2)-fuzzy subsets, then we call *τ* the discrete (3, 2)-fuzzy topology on *X*.

**Example 4 .**

Let *τ*={1_*X*_, 0_*X*_, *D*_1_, *D*_2_, *D*_3_, *D*_4_, *D*_5_} be the family of (3, 2)-fuzzy subsets of *X*={*x*_1_, *x*_2_}, where

(11) $$\begin{array}{c} D_{1}=\left\{\langle x_{1},\alpha_{D_{1}}\left(x_{1}\right)=0.8,\beta_{D_{1}}\left(x_{1}\right)=0.62\rangle ,\langle x_{2},\alpha_{D_{1}}\left(x_{2}\right)=0.81,\beta_{D_{1}}\left(x_{2}\right)=0.61\rangle \right\},\\ D_{2}=\left\{\langle x_{1},\alpha_{D_{2}}\left(x_{1}\right)=0.83,\beta_{D_{2}}\left(x_{1}\right)=0.53\rangle ,\langle x_{2},\alpha_{D_{2}}\left(x_{2}\right)=0.82,\beta_{D_{2}}\left(x_{2}\right)=0.62\rangle \right\},\\ D_{3}=\left\{\langle x_{1},\alpha_{D_{3}}\left(x_{1}\right)=0.79,\beta_{D_{3}}\left(x_{1}\right)=0.63\rangle ,\langle x_{2},\alpha_{D_{3}}\left(x_{2}\right)=8,\beta_{D_{3}}\left(x_{2}\right)=0.63\rangle \right\},\\ D_{4}=\left\{\langle x_{1},\alpha_{D_{4}}\left(x_{1}\right)=0.83,\beta_{D_{4}}\left(x_{1}\right)=0.53\rangle ,\langle x_{2},\alpha_{D_{4}}\left(x_{2}\right)=0.82,\beta_{D_{4}}\left(x_{2}\right)=0.61\rangle \right\},\\ D_{5}=\left\{\langle x_{1},\alpha_{D_{5}}\left(x_{1}\right)=0.8,\beta_{D_{5}}\left(1\right)=0.62\rangle ,\langle x_{2},\alpha_{D_{5}}\left(x_{2}\right)=0.81,\beta_{D_{5}}\left(x_{2}\right)=0.62\rangle \right\}. \end{array}$$

Hence, *τ* is (3, 2)-fuzzy topology on *X*.

**Remark 3 .**

We showed that every fuzzy set *D* on a set *X* is a (3, 2)-fuzzy set having the form *D*={〈*r*, *α*_*D*_(*r*), 1 − *α*_*D*_(*r*)〉 :  *r* ∈ *X*}. Then, every fuzzy topological space (*X*, *τ*_1_) in the sense of Chang is obviously a (3, 2)-fuzzy topological space in the form *τ*={*D* :  *α*_*D*_ ∈ *τ*_1_} whenever we identify a fuzzy set in *X* whose membership function is *α*_*D*_ with its counterpart *D*={〈*r*, *α*_*D*_(*r*), 1 − *α*_*D*_(*r*)〉 :  *r* ∈ *X*}. Similarly, one can note that every intuitionistic fuzzy topology (Pythagorean fuzzy topology) is (3, 2)-fuzzy topology. The following examples explain this note.

**Example 5 .**

Consider *τ*={1_*X*_, 0_*X*_, *D*_1_, *D*_2_} as family of fuzzy subsets of *X*={*x*}, where

(12) $$\begin{array}{c} 1_{X}=\left\{\langle c,\alpha_{1_{X}}\left(x\right)=1,1-\alpha_{1_{X}}\left(x\right)=\beta_{1_{X}}\left(x\right)=0\rangle \right\},\\ 0_{X}=\left\{\langle c,\alpha_{0_{X}}\left(x\right)=0,1-\alpha_{0_{X}}\left(x\right)=\beta_{0_{X}}\left(x\right)=1\rangle \right\},\\ D_{1}=\left\{\langle c,\alpha_{D_{1}}\left(x\right)=0.7,1-\alpha_{D_{1}}\left(x\right)=\beta_{D_{1}}\left(x\right)=0.3\rangle \right\},\\ D_{2}=\left\{\langle c,\alpha_{D_{2}}\left(x\right)=0.2,1-\alpha_{D_{2}}\left(x\right)=\beta_{D_{2}}\left(x\right)=0.8\rangle \right\}. \end{array}$$

Then, *τ* is fuzzy topology on *X*, and hence it is (3, 2)-fuzzy topology.

**Example 6 .**

Let *τ*={1_*X*_, 0_*X*_, *D*_1_, *D*_2_} be the family of (3, 2)-fuzzy subsets on *X*={*x*_1_, *x*_2_} where

(13) $$\begin{array}{c} D_{1}=\left\{\langle x_{1},\alpha_{D_{1}}\left(x_{1}\right)=0.76,\beta_{D_{1}}\left(x_{1}\right)=0.74\rangle ,\langle x_{2},\alpha_{D_{1}}\left(x_{2}\right)=0.6,\beta_{D_{1}}\left(x_{2}\right)=0.83\rangle \right\},\\ D_{2}=\left\{\langle x_{1},\alpha_{D_{2}}\left(x_{1}\right)=0.75,\beta_{D_{2}}\left(x_{1}\right)=0.74\rangle ,\langle x_{2},\alpha_{D_{2}}\left(x_{2}\right)=0.59,\beta_{D_{2}}\left(x_{2}\right)=0.83\rangle \right\}. \end{array}$$

Hence, *τ* is (3, 2)-fuzzy topology. On the other hand, *τ* is neither intuitionistic fuzzy topology nor Pythagorean fuzzy topology.

**Definition 6 .**

Let (*X*, *τ*) be a (3, 2)-fuzzy topological space and *D*={〈*x*, *α*_*D*_(*x*), *β*_*D*_(*x*)〉 :  *x* ∈ *X*} be a (3, 2)-FS in *X*. Then, the (3, 2)-fuzzy interior and (3, 2)-fuzzy closure of *D* are, respectively, defined by

1. cl(*D*)=∩{*H* : *H* is a closed (3, 2)-FS in *X* and *D* ⊂ *H*}.
2. int(*D*)=∪{*G* : *G* is an open (3, 2)-FS in *X* and *G* ⊂ *D*}.

**Remark 4 .**

Let (*X*, *τ*) be a (3, 2)-fuzzy topological space and *D* be any (3, 2)-FS in *X*. Then,

1. int(*D*) is an open (3, 2)-FS.
2. cl(*D*) is a closed (3, 2)-FS.
3. int(1_*X*_)=cl(1_*X*_)=1_*X*_ and int(0_*X*_)=cl(0_*X*_)=0_*X*_.

**Example 7 .**

Consider the (3, 2)-fuzzy topological space (*X*, *τ*) in Example 4. If *D*={〈*c*_1_, 0.67, 0.81〉, 〈*c*_2_, 0.75, 0.74〉}, then int(*D*)=0_*X*_ and cl(*D*)=1_*X*_.

**Theorem 5 .**

Let (*X*, *τ*) be a (3, 2)-fuzzy topological space and *D*_1_, *D*_2_ be (3, 2)-FSs in *X*. Then, the following properties hold:

1. int(*D*_1_) ⊂ *D*_1_ and *D*_1_ ⊂ cl(*D*_1_).
2. If *D*_1_ ⊂ *D*_2_, then int(*D*_1_) ⊂ int(*D*_2_) and cl(*D*_1_) ⊂ cl(*D*_2_).
3. *D*_1_ is an open (3, 2)-FS if and only if *D*_1_=int(*D*_1_).
4. *D*_1_ is a closed (3, 2)-FS if and only if *D*_1_=cl(*D*_1_).

**Proof**

(1) and (2) are obvious.

(3) and (4) follow from Definition 6.

**Corollary 1 .**

Let (*X*, *τ*) be a (3, 2)-fuzzy topological space and *D*_1_, *D*_2_ be (3, 2)-FSs in *X*. Then, the following properties hold:

1. int(*D*_1_) ∪ int(*D*_2_) ⊂ int(*D*_1_ ∪ *D*_2_).
2. cl(*D*_1_∩*D*_2_) ⊂ cl(*D*_1_)∩cl(*D*_2_).
3. int(*D*_1_∩*D*_2_)=int(*D*_1_)∩int(*D*_2_).
4. cl(*D*_1_) ∪ cl(*D*_2_)=cl(*D*_1_ ∪ *D*_2_).

**Proof**

(1) and (2) follows from (1) of the above theorem.

(3): since int(*D*_1_∩*D*_2_) ⊂ int(*D*_1_) and int(*D*_1_∩*D*_2_) ⊂ int(*D*_2_), we obtain int(*D*_1_∩*D*_2_) ⊂ int(*D*_1_)∩int(*D*_2_). On the other hand, from the facts int(*D*_1_) ⊂ *D*_1_ and int(*D*_2_) ⊂ *D*_2_, we have int(*D*_1_)∩int(*D*_2_) ⊂ *D*_1_∩*D*_2_ and int(*D*_1_)∩int(*D*_2_) ∈ *τ*; we see that int(*D*_1_)∩int(*D*_2_) ⊂ int(*D*_1_∩*D*_2_), and hence int(*D*_1_∩*D*_2_)=int(*D*_1_)∩int(*D*_2_).

(4) can be proved similar to (3).

**Theorem 6 .**

Let (*X*, *τ*) be a (3, 2)-fuzzy topological space and *D* be (3, 2)-FS in *X*. Then, the following properties hold:

1. cl(*D*^*c*^)=int(*D*)^*c*^.
2. int(*D*^*c*^)=cl(*D*)^*c*^.
3. cl(*D*^*c*^)^*c*^=int(*D*).
4. int(*D*^*c*^)^*c*^=cl(*D*).

**Proof**

We only prove (1); the other parts can be proved similarly.

Let *D*={〈*x*, *α*_*D*_(*x*), *β*_*D*_(*x*)〉 :  *x* ∈ *X*} and suppose that the family of open (3, 2)-fuzzy sets contained in *D* is indexed by the family {〈*x*, *α*_*U*_*i*__(*x*), *β*_*U*_*i*__(*x*)〉 :  *i* ∈ *J*}. Then, int(*D*)={〈*x*, ∨*α*_*U*_*i*__(*x*), ∧*β*_*U*_*i*__(*x*)〉}. Therefore, int(*D*)^*c*^={〈*x*, ∧*β*_*U*_*i*__(*x*), ∨*α*_*U*_*i*__(*x*)〉}. Now, *D*^*c*^={〈*x*, *β*_*D*_(*x*), *α*_*D*_(*x*)〉} such that *α*_*U*_*i*__ ≤ *α*_*D*_, *β*_*U*_*i*__ ≥ *β*_*D*_ for each *i* ∈ *J*. This implies that {〈*x*, *β*_*U*_*i*__(*x*), *α*_*U*_*i*__(*x*)〉 :  *i* ∈ *J*} is the family of all closed (3, 2)-fuzzy sets containing *D*^*c*^. That is, cl(*D*^*c*^)={〈*x*, ∧*β*_*U*_*i*__(*x*), ∨*α*_*U*_*i*__(*x*)〉}. Hence, cl(*D*^*c*^)=int(*D*)^*c*^.

### 3.2. (3, 2)-Fuzzy Continuous Maps

**Definition 7 .**

Let *f* : *X*⟶*Y* be a map and *A* and *B* be (3, 2)-fuzzy subsets of *X* and *Y*, respectively. The functions of membership and non-membership of the image of *A*, denoted by *f*[*A*], are, respectively, calculated by

(14) $$\begin{array}{c} \alpha_{f\left[A\right]}\left(y\right)≔\left\{\begin{array}{cc} \underset{z\in f^{-1}\left(y\right)}{\sup}\alpha_{A}\left(z\right), & \text{if }f^{-1}\left(y\right)\neq \phi ,\\ 0, & \text{otherwise}, \end{array}\right.\\ \beta_{f\left[A\right]}\left(y\right)≔\left\{\begin{array}{cc} \underset{z\in f^{-1}\left(y\right)}{\inf}\beta_{A}\left(z\right), & \text{if }f^{-1}\left(y\right)\neq \phi ,\\ 1, & \text{otherwise}. \end{array}\right. \end{array}$$

The functions of membership and non-membership of preimage of *B*, denoted by *f*^−1^[*B*], are, respectively, calculated by

(15) $$\begin{array}{c} \alpha_{f^{-1}\left[B\right]}\left(x\right)≔\alpha_{B}\left(f\left(x\right)\right),\\ \beta_{f^{-1}\left[B\right]}\left(x\right)≔\beta_{B}\left(f\left(x\right)\right). \end{array}$$

**Remark 5 .**

To show that *f*[*A*] and *f*^−1^[*B*] are (3, 2)-fuzzy subsets, consider *γ*_*A*_(*z*))^5^=*α*_*A*_(*z*))^3^+(*β*_*A*_(*z*))^2^. If *f*^−1^(*y*) is non-empty, then we obtain

(16) $$\begin{array}{c} {\left(\alpha_{f\left[A\right]}\left(y\right)\right)}^{3}+{\left(\beta_{f\left[A\right]}\left(y\right)\right)}^{2}={\left(\underset{z\in f^{-1}\left(y\right)}{\sup}\alpha_{A}\left(z\right)\right)}^{3}+{\left(\underset{z\in f^{-1}\left(y\right)}{\inf}\beta_{A}\left(z\right)\right)}^{2}\\ =\underset{z\in f^{-1}\left(y\right)}{\sup}{\left(\alpha_{A}\left(z\right)\right)}^{3}+\underset{z\in f^{-1}\left(y\right)}{\inf}{\left(\beta_{A}\left(z\right)\right)}^{2}\\ =\underset{z\in f^{-1}\left(y\right)}{\sup}\left({\left(\gamma_{A}\left(z\right)\right)}^{5}-{\left(\beta_{A}\left(z\right)\right)}^{2}\right)+\underset{z\in f^{-1}\left(y\right)}{\inf}{\left(\beta_{A}\left(z\right)\right)}^{2}\\ \leq \underset{z\in f^{-1}\left(y\right)}{\sup}\left(1-{\left(\beta_{A}\left(z\right)\right)}^{2}\right)+\underset{z\in f^{-1}\left(y\right)}{\inf}{\left(\beta_{A}\left(z\right)\right)}^{2}=1. \end{array}$$

In contrast, *f*^−1^(*y*)=*ϕ* leads to the fact that (*α*_*f*[*A*]_(*y*))^3^+(*β*_*f*[*A*]_(*y*))^2^=1.

It is easy to prove the case of *f*^−1^[*B*].

**Theorem 7 .**

Let *f* : *X*⟶*Y* be a map s.t. *A* and *B* are (3, 2)-fuzzy subsets of *X* and *Y*, respectively. Then, we have

1. *f*^−1^[*B*^*c*^]=*f*^−1^[*B*]^*c*^.
2. *f*[*A*]^*c*^⊆*f*[*A*^*c*^].
3. If *B*_1_⊆*B*_2_, then *f*^−1^[*B*_1_]⊆*f*^−1^[*B*_2_] where *B*_1_ and *B*_2_ are (3, 2)-fuzzy subsets of *Y*.
4. If *A*_1_⊆*A*_2_, then *f*[*A*_1_]⊆*f*[*A*_2_] where *A*_1_ and *A*_2_ are (3, 2)-fuzzy subsets of *X*.
5. *f*[*f*^−1^[*B*]]⊆*B*.
6. *A*⊆*f*^−1^[*f*[*A*]].

**Proof**

(1) Consider *v* ∈ *X* and let *B* be a (3, 2)-fuzzy subset of *Y*. Then,
  (17) $$\begin{array}{c} \alpha_{f^{-1}\left[B^{c}\right]}\left(v\right)=\alpha_{B^{c}}\left(f\left(v\right)\right)=\beta_{B}\left(f\left(v\right)\right)=\beta_{f^{-1}\left[B\right]}\left(v\right)=\alpha_{f^{-1}{\left[B\right]}^{c}}\left(v\right). \end{array}$$
Similarly, one can have *β*_*f*^−1^[*B*^*c*^]_(*v*)=*β*_*f*^−1^[*B*]^*c*^_(*v*). Therefore, *f*^−1^[*B*^*c*^]=*f*^−1^[*B*]^*c*^, as required.
(2) For any *w* ∈ *Y* such that *f*^−1^(*w*) ≠ *ϕ* and for any (3, 2)-fuzzy subset *A* of *X*, we can write
  (18) $$\begin{array}{c} {\left(\gamma_{f\left[A\right]}\left(w\right)\right)}^{5}={\left(\alpha_{f\left[A\right]}\left(w\right)\right)}^{3}+{\left(\beta_{f\left[A\right]}\left(w\right)\right)}^{2}\\ =\underset{z\in f^{-1}\left(w\right)}{\sup}{\left(\alpha_{A}\left(z\right)\right)}^{3}+\underset{z\in f^{-1}\left(w\right)}{\inf}{\left(\beta_{A}\left(z\right)\right)}^{2}\\ =\underset{z\in f^{-1}\left(w\right)}{\sup}\left({\left(\gamma_{A}\left(z\right)\right)}^{5}-{\left(\beta_{A}\left(z\right)\right)}^{2}\right)+\underset{z\in f^{-1}\left(w\right)}{\inf}{\left(\beta_{A}\left(z\right)\right)}^{2}\\ \leq \underset{z\in f^{-1}\left(w\right)}{\sup}\left({\left(\gamma_{A}\left(z\right)\right)}^{5}\right)-\underset{z\in f^{-1}\left(w\right)}{\inf}{\left(\beta_{A}\left(z\right)\right)}^{2}+\underset{z\in f^{-1}\left(w\right)}{\inf}{\left(\beta_{A}\left(z\right)\right)}^{2}\\ =\underset{z\in f^{-1}\left(w\right)}{\sup}\left({\left(\gamma_{A}\left(z\right)\right)}^{5}\right). \end{array}$$
Now from (18), we have
  (19) $$\begin{array}{c} \alpha_{f\left[A^{c}\right]}\left(w\right)=\underset{z\in f^{-1}\left(w\right)}{\sup}\alpha_{A^{c}}\left(z\right)\\ =\underset{z\in f^{-1}\left(w\right)}{\sup}\beta_{A}\left(z\right)\\ =\underset{z\in f^{-1}\left(w\right)}{\sup}\sqrt{{\left(\gamma_{A}\left(z\right)\right)}^{5}-{\left(\alpha_{A}\left(z\right)\right)}^{3}}\\ \geq \sqrt{\underset{z\in f^{-1}\left(w\right)}{\sup}{\left(\gamma_{A}\left(z\right)\right)}^{5}-\underset{z\in f^{-1}\left(w\right)}{\sup}{\left(\alpha_{A}\left(z\right)\right)}^{3}}\\ \geq \sqrt{{\left(\gamma_{f\left[A\right]}\left(w\right)\right)}^{5}-{\left(\alpha_{f\left[A\right]}\left(w\right)\right)}^{3}}\\ =\beta_{f\left[A\right]}\left(w\right)\\ =\alpha_{f{\left[A\right]}^{c}}\left(w\right). \end{array}$$
The proof is easy when *f*^−1^(*w*)=*ϕ*. Following a similar technique, we obtain *β*_*f*[*A*^*c*^]_(*w*) ≤ *β*_*f*[*A*]^*c*^_(*w*), which means that *f*[*A*]^*c*^⊆*f*[*A*^*c*^].
(3) Assume that *B*_1_⊆*B*_2_. Then, for each *v* ∈ *X*, *α*_*f*^−1^[*B*_1_]_(*v*)=*α*_*B*_1__(*f*(*v*)) ≤ *α*_*B*_2__(*f*(*v*))=*α*_*f*^−1^[*B*_2_]_(*v*). Also, *β*_*f*^−1^[*B*_1_]_(*v*) ≥ *β*_*f*^−1^[*B*_2_]_(*v*). Hence, we obtain the desired result.
(4) Assume that *A*_1_⊆*A*_2_ and *w* ∈ *Y*. The proof is easy when *f*(*w*)=*ϕ*. So, presume that *f*(*w*) ≠ *ϕ*. Then,
  (20) $$\begin{array}{c} \alpha_{f\left[A_{1}\right]}\left(w\right)=\underset{z\in f^{-1}\left(w\right)}{\sup}\alpha_{A_{1}}\left(z\right)\leq \underset{z\in f^{-1}\left(w\right)}{\sup}\alpha_{A_{2}}\left(z\right)=\alpha_{f\left[A_{2}\right]}\left(w\right). \end{array}$$
Thus, *α*_*f*[*A*_1_]_ ≤ *α*_*f*[*A*_2_]_ follows. Similarly, we have *β*_*f*[*A*_1_]_ ≥ *β*_*f*[*A*_2_]_.
(5) For any *w* ∈ *Y* s.t. *f*(*w*) ≠ *ϕ*, we find that
  (21) $$\begin{array}{c} \alpha_{f\left[f^{-1}\left[B\right]\right]}\left(w\right)=\underset{z\in f^{-1}\left(w\right)}{\sup}\alpha_{f^{-1}\left[B\right]}\left(z\right)=\underset{z\in f^{-1}\left(w\right)}{\sup}\alpha_{B}\left(f\left(z\right)\right)\leq \alpha_{B}\left(w\right). \end{array}$$
On the other hand, we have *α*_*f*[*f*^−1^[*B*]]_(*w*)=0 ≤ *α*_*B*_(*w*) when *f*(*w*)=*ϕ*. Similarly, we have *β*_*f*[*f*^−1^[*B*]]_(*w*)=0 ≥ *β*_*B*_(*w*).
(6) For any *v* ∈ *X*, we have
  (22) $$\begin{array}{c} \alpha_{f^{-1}\left[f\left[A\right]\right]}\left(v\right)=\alpha_{f\left[A\right]}\left(f\left(v\right)\right)=\underset{z\in f^{-1}\left(f\left(v\right)\right)}{\sup}\alpha_{A}\left(z\right)\geq \alpha_{A}\left(v\right). \end{array}$$
Similarly, we have *β*_*f*^−1^[*f*[*A*]]_ ≤ *β*_*A*_.

The proof of the following result is easy, and hence it is omitted.

**Theorem 8 .**

Let *X* and *Y* be two non-empty sets and *f* : *X*⟶*Y* be a map. Then, the following statements are true:

1. *f*[∪_*i*∈*I*_*A*_*i*_]=∪_*i*∈*I*_*f*[*A*_*i*_] for any (3, 2)-fuzzy subset *A*_*i*_ of *X*.
2. *f*^−1^[∪_*i*∈*I*_*B*_*i*_]=∪_*i*∈*I*_*f*^−1^[*B*_*i*_] for any (3, 2)-fuzzy subset *B*_*i*_ of *Y*.
3. *f*[*A*_1_∩*A*_2_] ⊂ *f*[*A*_1_]∩*f*[*A*_2_] for any two (3, 2)-fuzzy subsets *A*_1_ and *A*_2_ of *X*.
4. *f*^−1^[∩_*i*∈*I*_*B*_*i*_]=∩_*i*∈*I*_*f*^−1^(*B*_*i*_) for any (3, 2)-fuzzy subset *B*_*i*_ of *Y*.

**Definition 8 .**

In a (3, 2)-fuzzy topological space, consider that *A* and *U* are two (3, 2)-fuzzy subsets. We call *U* a neighborhood of *A*, briefly nbd, if there exists an open (3, 2)-fuzzy subset *E* such that *A*⊆*E*⊆*U*.

**Theorem 9 .**

A (3, 2)-fuzzy subset *A* is open iff it contains a nbd of its each subset.

**Proof**

The proof is easy.

**Definition 9 .**

A map *f* : (*X*, *τ*_1_)⟶(*Y*, *τ*_2_) is said to be (3, 2)-fuzzy continuous if for any (3, 2)-fuzzy subset *A* of *X* and for any nbd *V* of *f*[*A*] there is a nbd *U* of *A* s.t. *f*[*U*]⊆*V*.

**Theorem 10 .**

The following statements are equivalent for a map *f* : (*X*, *τ*_1_)⟶(*Y*, *τ*_2_):

1. *f* is (3, 2)-fuzzy continuous.
2. For each (3, 2)-FS *A* of *X* and each nbd *V* of *f*[*A*], there is a nbd *U* of *A* s.t. for each *B*⊆*U*, we obtain *f*[*B*]⊆*V*.
3. For each (3, 2)-FS *A* of *X* and for each nbd *V* of *f*[*A*], there is a nbd *U* of *A* s.t. *U*⊆*f*^−1^[*V*].
4. For each (3, 2)-FS *A* of *X* and for each nbd *V* of *f*[*A*], *f*^−1^[*V*] is a nbd of *A*.

**Proof**

 
(1)⇒(2): let *f* be a (3, 2)-fuzzy continuous map. Consider *A* as a (3, 2)-FS of *X* and *V* as a nbd of *f*[*A*]. Then, there is a nbd *U* of *A* s.t. *f*[*U*]⊆*V*. If *B*⊆*U*, we obtain *f*[*B*]⊆*f*[*U*]⊆*V*.
 
(2)⇒(3): assume *A* as a (3, 2)-FS of *X* and *V* as a nbd of *f*[*A*]. According to (2), there is a nbd *U* of *A* s.t. for each *B*⊆*U*, we find *f*[*B*]⊆*V*. Therefore, *B*⊆*f*^−1^[*f*[*B*]]⊆*f*^−1^[*V*]. Since *B* is chosen arbitrarily, *U*⊆*f*^−1^[*V*].
 
(3)⇒(4): presume *A* is a (3, 2)-FS of *X* and *V* is a nbd of *f*[*A*]. According to (3), there is a nbd *U* of *A* s.t. *U*⊆*f*^−1^[*V*]. Since *U* is a nbd of *A*, there is an open (3, 2)-FS *K* of *X* s.t. *A*⊆*K*⊆*U*. On the other hand, we obtain *A*⊆*K*⊆*f*^−1^[*V*] because *U*⊆*f*^−1^[*V*]. This means that *f*^−1^[*V*] is a nbd of *A*.
 
(4)⇒(1): suppose that *A* is a (3, 2)-FS of *X* and *V* is a nbd of *f*[*A*]. By hypothesis, *f*^−1^[*V*] is a nbd of *A*. So, there is an open (3, 2)-FS *K* of *X* s.t. *A*⊆*K*⊆*f*^−1^[*V*] which means *f*[*K*]⊆*f*[*f*^−1^[*V*]]⊆*V*. Moreover, *K* is an open (3, 2)-FS, so it is a nbd of *A*. Hence, we obtain the proof that *f* is (3, 2)-fuzzy continuous.

**Theorem 11 .**

A map *f* : (*X*, *τ*_1_)⟶(*Y*, *τ*_2_) is (3, 2)-fuzzy continuous iff *f*^−1^[*B*] is an open (3, 2)-FS of *X* for each open (3, 2)-FS *B* of *Y*.

**Proof**

Necessity: presume *f* as a (3, 2)-fuzzy continuous map. Consider an open (3, 2)-FS *B* of *Y* s.t. *A*⊆*f*^−1^[*B*]. This directly gives that *f*[*A*]⊆*B*. It follows from Theorem 9 that there is a nbd *V* of *f*[*A*] satisfying *V*⊆*B*. Now, *f* is (3, 2)-fuzzy continuous, so by (4) of Theorem 10, we obtain that *f*^−1^[*V*] is a nbd of *A*. Also, it follows from (3) of Theorem 7 that *f*^−1^[*V*]⊆*f*^−1^[*B*]. So, *f*^−1^[*B*] is a nbd of *A*. Since *A* is an arbitrary subset of *f*^−1^[*B*], then by Theorem 9, the (3, 2)-FS *f*^−1^[*B*] is open.

#### 3.2.1. Sufficiency

Presume *A* is a (3, 2)-FS of *X* and *V* is a nbd of *f*[*A*]. Then, *τ*_2_ contains a (3, 2)-FS *L* of s.t. *f*[*A*]⊆*L*⊆*V*. By hypothesis, *f*^−1^[*L*] is an open (3, 2)-FS. Also, we have *A*⊆*f*^−1^[*f*[*A*]]⊆*f*^−1^[*L*]⊆*f*^−1^[*V*]. Thus, *f*^−1^[*V*] is a nbd of *A* which demonstrates that *f* is (3, 2)-fuzzy continuous.

We build the following two examples such that the first one provides a (3, 2)-fuzzy continuous map, whereas the second one presents a fuzzy map that is not (3, 2)-fuzzy continuous.

**Example 8 .**

Consider *X*={*a*_1_, *a*_2_} with the (3, 2)-fuzzy topology *τ*_1_={1_*X*_, 0_*X*_, *A*_1_} and *Y*={*b*_1_, *b*_2_} with the (3, 2)-fuzzy topology *τ*_2_={1_*Y*_, 0_*Y*_, *B*_1_}, where

(23) $$\begin{array}{c} A_{1}=\left\{\langle a_{1},0.7,0.78\rangle ,\langle a_{2},0.9,0.5\rangle \right\},\\ B_{1}=\left\{\langle b_{1},0.9,0.5\rangle ,\langle b_{2},0.7,0.78\rangle \right\}. \end{array}$$

Let *f* : *X*⟶*Y* be defined as follows:

(24) $$\begin{array}{c} f\left(x\right)=\left\{\begin{array}{cc} b_{2}, & \text{if }x=a_{1},\\ b_{1}, & \text{if }x=a_{2}. \end{array}\right. \end{array}$$

Since 1_*Y*_, 0_*Y*_, and *B*_1_ are open (3, 2)-fuzzy subsets of *Y*, then

(25) $$\begin{array}{c} f^{-1}\left[1_{Y}\right]=\left\{\langle a_{1},1,0\rangle ,\langle a_{2},1,0\rangle \right\},\\ f^{-1}\left[0_{Y}\right]=\left\{\langle a_{1},0,1\rangle ,\langle a_{2},0,1\rangle \right\},\\ f^{-1}\left[B_{1}\right]=\left\{\langle a_{1},0.7,0.78\rangle ,\langle a_{2},0.9,0.5\rangle \right\} \end{array}$$

are open (3, 2)-fuzzy subsets of *X*. Thus, *f* is (3, 2)-fuzzy continuous.

**Example 9 .**

Consider *X*={*a*_1_, *a*_2_} with the (3, 2)-fuzzy topology *τ*_1_={1_*X*_, 0_*X*_} and *Y*={*b*_1_, *b*_2_} with the (3, 2)-fuzzy topology *τ*_2_={1_*Y*_, 0_*Y*_, *B*_1_}, where *B*_1_={〈*b*_1_, 0.82, 0.62〉, 〈*b*_2_, 0.52, 0.90〉}.

Let *f* : *X*⟶*Y* be defined as follows:

(26) $$\begin{array}{c} f\left(x\right)=\left\{\begin{array}{cc} b_{1}, & \text{if }x=a_{1},\\ b_{2}, & \text{if }x=a_{2}. \end{array}\right. \end{array}$$

Since *B*_1_ is an open (3, 2)-fuzzy subset of *Y*, but *f*^−1^[*B*_1_]={〈*a*_1_, 0.82, 0.62〉, 〈*a*_2_, 0.52, 0.90〉} is not an open (3, 2)-fuzzy subset of *X*, *f* is not (3, 2)-fuzzy continuous.

**Theorem 12 .**

The following are equivalent to each other:

1. *f* : (*X*, *τ*_1_)⟶(*Y*, *τ*_2_) is (3, 2)-fuzzy continuous.
2. For each closed (3, 2)-fuzzy subset *B* of *Y* we have that *f*^−1^[*B*] is a closed (3, 2)-fuzzy subset of *X*.
3. cl(*f*^−1^[*B*])⊆*f*^−1^[cl(*B*)] for each (3, 2)-fuzzy set in *Y*.
4. *f*^−1^[int(*B*)]⊆int(*f*^−1^[*B*]) for each (3, 2)-fuzzy set in *Y*.

**Proof**

They can be easily proved using Theorems 6, 7, and 11.

**Theorem 13 .**

Let (*Y*, *τ*) be a (3, 2)-fuzzy topological space and *f* : *X*⟶*Y* be a map. Then, there is a coarsest (3, 2)-fuzzy topology *τ*_1_ over *X* such that *f* is (3, 2)-fuzzy continuous.

**Proof**

Let us define a class of (3, 2)-fuzzy subsets *τ*_1_ of *X* by

(27) $$\begin{array}{c} \tau_{1}≔\left\{f^{-1}\left[V\right]:\;V\in \tau \right\}. \end{array}$$

We prove that *τ*_1_ is the coarsest (3, 2)-fuzzy topology over *X* such that *f* is (3, 2)-fuzzy continuous.

(1) We can write for any *x* ∈ *X* that
  (28) $$\begin{array}{c} \alpha_{f^{-1}\left[0_{Y}\right]}\left(x\right)=\alpha_{0_{Y}}\left(f\left(x\right)\right)=0=\alpha_{0_{X}}\left(x\right). \end{array}$$
Similarly, we immediately have *β*_*f*^−1^[0_*Y*_]_(*x*)=*β*_0_*X*__(*x*) for any *x* ∈ *X* which implies *f*^−1^[0_*Y*_]=0_*X*_. Now, as 0_*Y*_ ∈ *τ*, we have 0_*X*_=*f*^−1^[0_*Y*_] ∈ *τ*_1_. In a similar manner, it is easy to see that 1_*X*_=*f*^−1^[1_*Y*_] ∈ *τ*_1_.
(2) Assume that *D*_1_, *D*_2_ ∈ *τ*_1_. Then, for *i*=1,2, there exists *B*_*i*_ ∈ *τ* such that *f*^−1^[*B*_*i*_]=*D*_*i*_ which implies *α*_*f*^−1^[*B*_*i*_]_=*α*_*D*_*i*__ and *β*_*f*^−1^[*B*_*i*_]_=*β*_*D*_*i*__. Thus, we obtain for any *x* ∈ *X* that
  (29) $$\begin{array}{c} \alpha_{D_{1}\cap D_{2}}\left(x\right)=\min\left\{\left\{\alpha_{D_{1}}\left(x\right),\alpha_{D_{2}}\left(x\right)\right\}\right\}=\min\left\{\alpha_{f^{-1}\left[B_{1}\right]}\left(x\right),\alpha_{f^{-1}\left[B_{2}\right]}\left(x\right)\right\}=\min\left\{\alpha_{B_{1}}\left(f\left(x\right)\right),\alpha_{B_{2}}\left(f\left(x\right)\right)\right\}\\ =\alpha_{B_{1}\cap B_{2}}\left(f\left(x\right)\right)=\alpha_{f^{-1}\left[B_{1}\cap B_{2}\right]}\left(x\right). \end{array}$$
Similarly, it is not difficult to see that *β*_*D*_1_∩*D*_2__=*β*_*f*^−1^[*B*_1_∩*B*_2_]_. Hence, we get *D*_1_∩*D*_2_ ∈ *τ*_1_.
(3) Assume that {*D*_*i*_}_*i*∈*I*_ is an arbitrary subfamily of *τ*_1_. Then, for any *i* ∈ *I*, there exists *B*_*i*_ ∈ *τ*_1_ such that *f*^−1^[*B*_*i*_]=*D*_*i*_ which implies *α*_*f*^−1^[*B*_*i*_]_=*α*_*D*_*i*__ and *β*_*f*^−1^[*B*_*i*_]_=*β*_*D*_*i*__. Therefore, one can get for any *x* ∈ *X* that

(30) $$\begin{array}{c} \alpha_{\cup_{i\in I}D_{i}}\left(x\right)=\underset{i\in I^{\alpha }D_{i}}{\sup}\left(x\right)\\ =\underset{i\in I^{\alpha }f^{-1}\left[B_{i}\right]}{\sup}\left(x\right)\\ =\underset{i\in I^{\alpha }B_{i}}{\sup}\left(f\left(x\right)\right)\\ =\alpha_{\cup_{i\in I}B_{i}}\left(f\left(x\right)\right)\\ =\alpha_{f^{-1}\left[\cup_{i\in I}B_{i}\right]}\left(x\right). \end{array}$$

On the other hand, it is easy to see that *β*_∪_*i*∈*I*_*D*_*i*__=*β*_*f*^−1^[∪_*i*∈*I*_*B*_*i*_]_. Thus, we have ∪_*i*∈*I*_*D*_*i*_ ∈ *τ*_1_.

From Theorem 11, the (3, 2)-fuzzy continuity of *f* is trivial. Now, we prove that *τ*_1_ is the coarsest (3, 2)-fuzzy topology over *X* such that *f* is (3, 2)-fuzzy continuous. Let *τ*_2_⊆*τ*_1_ be a (3, 2)-fuzzy topology over *X* such that *f* is (3, 2)-fuzzy continuous. If *B* ∈ *τ*_1_, then there is *V* ∈ *τ* such that *f*^−1^[*V*]=*B*. Since *f* is (3, 2)-fuzzy continuous with respect to *τ*_2_, we have *B*=*f*^−1^[*V*] ∈ *τ*_2_. Hence, *τ*_2_=*τ*_1_, as required.

### 3.3. (3, 2)-Fuzzy Separation Axioms

Separation axioms are one of the most important and popular notions in topological studies. They have been studied and applied to model some real-life issues in soft setting as explained in [16, 17].

**Definition 10 .**

Let *X* ≠ ∅ and *x* ∈ *X* be a fixed element in *X*. Suppose that *r*_1_ ∈ (0,1] and *r*_2_ ∈ [0,1) are two fixed real numbers such that *r*_1_^3^+*r*_2_^2^ ≤ 1. Then, a (3, 2)-fuzzy point *p*_(*r*_1_, *r*_2_)_^*x*^={〈*x*, *α*_*p*_(*x*), *β*_*p*_(*x*)〉} is defined to be a (3, 2)-fuzzy set of *X* as follows.

(31) $$\begin{array}{c} p_{\left(r_{1},r_{2}\right)}^{x}\left(y\right)≔\left\{\begin{array}{cc} \left(r_{1},r_{2}\right), & \text{if }y=x,\\ \left(0,1\right), & \text{otherwise}, \end{array}\right. \end{array}$$

for *y* ∈ *X*. In this case, *x* is called the support of *p*_(*r*_1_, *r*_2_)_^*x*^. A (3, 2)-fuzzy point *p*_(*r*_1_, *r*_2_)_^*x*^ is said to belong to a (3, 2)-fuzzy set *D*={〈*x*, *α*_*D*_(*x*), *β*_*D*_(*x*)〉} of *X* denoted by *p*_(*r*_1_, *r*_2_)_^*x*^ ∈ *D* if *r*_1_ ≤ *α*_*D*_(*x*) and *r*_2_ ≥ *β*_*D*_(*x*). Two (3, 2)-fuzzy points are said to be distinct if their supports are distinct.

**Remark 6 .**

Let *D*_1_={〈*x*, *α*_*D*_1__(*x*), *β*_*D*_1__(*x*)〉} and *D*_2_={〈*x*, *α*_*D*_2__(*x*), *β*_*D*_2__(*x*)〉} be two (3, 2)-fuzzy sets of *X*. Then, *D*_1_⊆*D*_2_ if and only if *p*_(*r*_1_, *r*_2_)_^*x*^ ∈ *D*_1_ implies *p*_(*r*_1_, *r*_2_)_^*x*^ ∈ *D*_2_ for any (3, 2)-fuzzy point *p*_(*r*_1_, *r*_2_)_^*x*^ in *X*.

**Definition 11 .**

Let *r*_1_, *r*_3_ ∈ (0,1], *r*_2_, *r*_4_ ∈ [0,1), and *x*, *y* ∈ *X*. A (3, 2)-fuzzy topological space (*X*, *τ*) is said to be

(1) *T*_0_ if for each pair of distinct (3, 2)-fuzzy points *p*_(*r*_1_, *r*_2_)_^*x*^, *p*_(*r*_3_, *r*_4_)_^*y*^ in *X*, there exist two open (3, 2)-fuzzy sets *L* and *K* such that
  (32) $$\begin{array}{c} L=\left\{\langle x,1,0\rangle ,\langle y,0,1\rangle \right\},\\ \text{or }K=\left\{\langle x,0,1\rangle ,\langle y,1,0\rangle \right\}. \end{array}$$
(2) *T*_1_ if for each pair of distinct (3, 2)-fuzzy points *p*_(*r*_1_, *r*_2_)_^*x*^, *p*_(*r*_3_, *r*_4_)_^*y*^ in *X*, there exist two open (3, 2)-fuzzy sets *L* and *K* such that
  (33) $$\begin{array}{c} L=\left\{\langle x,1,0\rangle ,\langle y,0,1\rangle \right\},\\ K=\left\{\langle x,0,1\rangle ,\langle y,1,0\rangle \right\}. \end{array}$$

**Proposition 1 .**

Let (*X*, *τ*) be a (3, 2)-fuzzy topological space. If (*X*, *τ*) is *T*_1_, then (*X*, *τ*) is *T*_0_.

**Proof**

The proof is straightforward from Definition 11.

Here is an example which shows that the converse of above proposition is not true in general.

**Example 10 .**

Consider *X*={*c*_1_, *c*_2_} with the (3, 2)-fuzzy topology *τ*={1_*X*_, 0_*X*_, *D*}, where *D*={〈*c*_1_, 1,0〉, 〈*c*_2_, 0,1〉}. Then, (*X*, *τ*) is *T*_0_ but not *T*_1_ because there does not exist an open (3, 2)-fuzzy set *K* such that *K*={〈*x*, 0,1〉, 〈*y*, 1,0〉}.

**Theorem 14 .**

Let (*X*, *τ*) be a (3, 2)-fuzzy topological space, *r*_1_, *r*_3_ ∈ (0,1], and *r*_2_, *r*_4_ ∈ [0,1). If (*X*, *τ*) is *T*_0_, then for each pair of distinct (3, 2)-fuzzy points *p*_(*r*_1_, *r*_2_)_^*x*^, *p*_(*r*_3_, *r*_4_)_^*y*^ of *X*, cl(*p*_(*r*_1_, *r*_2_)_^*x*^) ≠ cl(*p*_(*r*_3_, *r*_4_)_^*y*^).

**Proof**

Let (*X*, *τ*) be *T*_0_ and *p*_(*r*_1_, *r*_2_)_^*x*^, *p*_(*r*_3_, *r*_4_)_^*y*^ be any two distinct (3, 2)-fuzzy points of *X*. Then, there exist two open (3, 2)-fuzzy sets *L* and *K* such that

(34) $$\begin{array}{c} L=\left\{\langle x,1,0\rangle ,\langle y,0,1\rangle \right\},\\ \text{or }K=\left\{\langle x,0,1\rangle ,\langle y,1,0\rangle \right\}. \end{array}$$

Let *L*={〈*x*, 1,0〉, 〈*y*, 0,1〉} exist. Then, *L*^*c*^={〈*x*, 0,1〉, 〈*y*, 1,0〉} is a closed (3, 2)-fuzzy set which does not contain *p*_(*r*_1_, *r*_2_)_^*x*^ but contains *p*_(*r*_3_, *r*_4_)_^*y*^. Since cl(*p*_(*r*_3_, *r*_4_)_^*y*^) is the smallest closed (3, 2)-fuzzy set containing *p*_(*r*_3_, *r*_4_)_^*y*^, then cl(*p*_(*r*_3_, *r*_4_)_^*y*^)⊆*L*^*c*^, and therefore *p*_(*r*_1_, *r*_2_)_^*x*^ ∉ cl(*p*_(*r*_3_, *r*_4_)_^*y*^). Consequently, cl(*p*_(*r*_1_, *r*_2_)_^*x*^) ≠ cl(*p*_(*r*_3_, *r*_4_)_^*y*^).

**Theorem 15 .**

Let (*X*, *τ*) be a (3, 2)-fuzzy topological space. If *p*_(1,0)_^*x*^ is closed (3, 2)-fuzzy set for every *x* ∈ *X*, then, (*X*, *τ*) is *T*_1_.

**Proof**

Suppose *p*_(1,0)_^*x*^ is a closed (3, 2)-fuzzy set for every *x* ∈ *X*. Let *p*_(*r*_1_, *r*_2_)_^*x*^, *p*_(*r*_3_, *r*_4_)_^*y*^ be any two distinct (3, 2)-fuzzy points of *X*; then, *x* ≠ *y* implies that *p*_(1,0)_^*x*^^*c*^ and *p*_(1,0)_^*y*^^*c*^ are two open (3, 2)-fuzzy sets such that

(35) $$\begin{array}{c} {p_{\left(1,0\right)}^{y}}^{c}=\left\{\langle x,1,0\rangle ,\langle y,0,1\rangle \right\},\\ {p_{\left(1,0\right)}^{x}}^{c}=\left\{\langle x,0,1\rangle ,\langle y,1,0\rangle \right\}. \end{array}$$

Thus, (*X*, *τ*) is *T*_1_.

## 4. (3, 2)-Fuzzy Relations

A relation is a mathematical description of a situation where certain elements of sets are related to one another in some way. The system of fuzzy relation equations was first studied by Sanchez [18–21], who used it in medical research. Biswas [22] defined the method of intuitionistic medical diagnosis which involves intuitionistic fuzzy relations. Kumar et al. [23] used the applications of intuitionistic fuzzy set theory in diagnosis of various types of diseases. The notion of max-min-max composite relation for Pythagorean fuzzy sets was studied by Ejegwa [24], and the approach was improved and applied to medical diagnosis.

In this section, we introduce the notions of max-min-max composite relation and improved composite relation for (3, 2)-FSs. Moreover, we provide a numerical example to elaborate on how we can apply the composite relations to obtain the optimal choices.

**Definition 12 .**

Let *X* and *Y* be two (crisp) sets. The (3, 2)-fuzzy relation *R* (briefly, (3, 2)-FR) from *X* to *Y* is a (3, 2)-FS of *X* × *Y* characterized by the degree of membership function *α*_*R*_ and degree of non-membership function *β*_*R*_. The (3, 2)-FR *R* from *X* to *Y* will be denoted by *R*(*X*⟶*Y*). If *D* is a (3, 2)-FS of *X*, then

(1) The max-min-max composition of the (3, 2)-FR *R*(*X*⟶*Y*) with *D* is a (3, 2)-FS *C* of *Y* denoted by *C* = *R o D* and is defined by
  (36) $$\begin{array}{c} \alpha_{RoD}\left(n\right)=\underset{m}{\vee }\left[\alpha_{D}\left(m\right)\wedge \alpha_{R}\left(m,n\right)\right],\\ \begin{array}{c} \beta_{RoD}\left(n\right)=\underset{m}{\wedge }\left[\beta_{D}\left(m\right)\vee \beta_{R}\left(m,n\right)\right],\\ \text{for all }n\in Y. \end{array} \end{array}$$
(2) The improved composite relation of *R*(*X*⟶*Y*) with *D* is a (3, 2)-FS *C* of *Y* denoted by *C* = *R o D*, such that

(37) $$\begin{array}{c} \alpha_{RoD}\left(n\right)=\underset{m}{\vee }\left[\frac{\alpha_{D}\left(m\right)+\alpha_{R}\left(m,n\right)}{2}\right],\\ \begin{array}{c} \beta_{RoD}\left(n\right)=\underset{m}{\wedge }\left[\frac{\beta_{D}\left(m\right)+\beta_{R}\left(m,n\right)}{2}\right],\\ \text{for all }n\in Y. \end{array} \end{array}$$

**Definition 13 .**

Let *Q*(*X*⟶*Y*) and *R*(*Y*⟶*Z*) be two (3, 2)-FRs. Then, for all (*m*, *r*) ∈ *X* × *Z* and *n* ∈ *Y*,

(1) The max-min-max composition *R o Q* is the (3, 2)-fuzzy relation from *X* to *Z* defined by
  (38) $$\begin{array}{c} \alpha_{RoQ}\left(m,r\right)=\underset{n}{\vee }\left[\alpha_{Q}\left(m,n\right)\wedge \alpha_{R}\left(n,r\right)\right],\\ \beta_{RoQ}\left(m,r\right)=\underset{n}{\wedge }\left[\beta_{Q}\left(m,n\right)\vee \beta_{R}\left(n,r\right)\right]. \end{array}$$
(2) The improved composite relation *R o Q* is the (3, 2)-fuzzy relation from *X* to *Z* such that

(39) $$\begin{array}{c} \alpha_{RoQ}\left(m,r\right)=\underset{n}{\vee }\left[\frac{\alpha_{Q}\left(m,n\right)+\alpha_{R}\left(n,r\right)}{2}\right],\\ \beta_{RoQ}\left(m,r\right)=\underset{n}{\wedge }\left[\frac{\beta_{Q}\left(m,n\right)+\beta_{R}\left(n,r\right)}{2}\right]. \end{array}$$

**Remark 7 .**

The improved composite and max-min-max composite relations for (3, 2)-fuzzy sets are calculated by the following:

(40) $$\begin{array}{c} S_{R}=\alpha_{RoQ}-\beta_{RoQ}\cdot \pi_{RoQ}. \end{array}$$

**Example 11 .**

Let *D*_1_ and *D*_2_ be two (3, 2)-fuzzy sets for *X*={*x*_1_, *x*_2_, *x*_3_, *x*_4_}. Assume that

(41) $$\begin{array}{c} D_{1}=\left\{\langle x_{1},0.8,0.61\rangle ,\langle x_{2},0.5,0.87\rangle ,\langle x_{3},0.85,0.55\rangle ,\langle x_{4},0.8,0.69\rangle \right\},\\ D_{2}=\left\{\langle x_{1},0.7,0.79\rangle ,\langle x_{2},0.78,0.73\rangle ,\langle x_{3},0.6,0.85\rangle ,\langle x_{4},0.89,0.54\rangle \right\}. \end{array}$$

By using Definitions 12 (1) and 13 (1), respectively, we find the max-min-max composite relation with application to *D*_1_ and *D*_2_ as follows:

(42) $$\begin{array}{c} \alpha_{C}\left(d_{1i},d_{2k}\right)=\underset{x_{j}}{\vee }\left[0.7,0.5,0.6,0.8\right]=0.8,\\ \beta_{C}\left(d_{1i},d_{2k}\right)=\underset{x_{j}}{\wedge }\left[0.79,0.87,0.85,0.69\right]=0.69. \end{array}$$

It is obvious that the minimum value of the membership values of the elements (that is, *x*_1_, *x*_2_, *x*_3_, *x*_4_) in *D*_1_ and *D*_2_, respectively, is 0.7, 0.5, 0.6, and 0.8. Also, the maximum value of the non-membership values of the elements (that is, *x*_1_, *x*_2_, *x*_3_, *x*_4_) in *D*_1_ and *D*_2_, respectively, is 0.79, 0.87, 0.85, and 0.69. From Remark 7, we can get

(43) $$\begin{array}{c} S_{R}=0.8-\left(0.69\right)\cdot \left(\sqrt{\left[5\right]}0.0119\right)\approx 0.52. \end{array}$$

Again, by using Definitions 12 (2) and 13 (2), respectively, we find the improved composite relation with application to *D*_1_ and *D*_2_ as follows:

(44) $$\begin{array}{c} \alpha_{C}\left(d_{1i},d_{2k}\right)=\underset{x_{j}}{\wedge }\left[0.75,0.64,0.725,0.845\right]=0.845,\\ \beta_{C}\left(d_{1i},d_{2k}\right)=\underset{x_{j}}{\wedge }\left[0.7,0.8,0.7,0.615\right]=0.615. \end{array}$$

From Remark 7, we can get

(45) $$\begin{array}{c} S_{R}=0.845-\left(0.615\right)\cdot \left(\sqrt{\left[5\right]}0.018423875\right)\approx 0.57. \end{array}$$

Hence, from (43) and (45), we obtain that the improved composite relation produces better relation with greater relational value when compared to max-min-max composite relation.

## 5. Application of (3, 2)-Fuzzy Sets

We localize the idea of (3, 2)-FR as follows.

Let *S*={*r*_1_,…, *r*_*l*_} be a finite set of subjects related to the colleges, *C*={*b*_1_,…, *b*_*m*_} be a finite set of colleges, and *A*={*t*_1_,…, *t*_*n*_} be a finite set of students. Suppose that we have two (3, 2)-FRs, *U*(*A*⟶*S*) and *R*(*S*⟶*C*), such that

(46) $$\begin{array}{c} U=\left\{\langle \left(t,r\right),\alpha_{U}\left(t,r\right),\beta_{U}\left(t,r\right)\rangle |\;\left(t,r\right)\in A\times S\right\},\\ R=\left\{\langle \left(r,b\right),\alpha_{R}\left(r,b\right),\beta_{R}\left(r,b\right)\rangle |\;\left(r,b\right)\in S\times C\right\}. \end{array}$$

where

 
*α*_*U*_(*t*, *r*) denotes the degree to which the student (*t*) passes the related subject requirement (*r*).
 
*β*_*U*_(*t*, *r*) denotes the degree to which the student (*t*) does not pass the related subject requirement (*r*).
 
*α*_*R*_(*r*, *b*) denotes the degree to which the related subject requirement (*r*) determines the college (*b*).
 
*β*_*R*_(*r*, *b*) denotes the degree to which the related subject requirement (*r*) does not determine the college (*b*).

*T*=*RoU* is the composition of *R* and *U*. This describes the state in which the students, *t*_*i*_, with respect to the related subject requirement, *r*_*j*_, fit the colleges, *b*_*k*_. Thus,

(47) $$\begin{array}{c} \alpha_{T}\left(t_{i},b_{k}\right)=\underset{r_{j}\in S}{\vee }\left[\alpha_{U}\left(t_{i},r_{j}\right)\wedge \alpha_{R}\left(r_{j},b_{k}\right)\right],\\ \beta_{T}\left(t_{i},b_{k}\right)=\underset{r_{j}\in S}{\wedge }\left[\beta_{U}\left(t_{i},r_{j}\right)\vee \beta_{R}\left(r_{j},b_{k}\right)\right], \end{array}$$

∀*t*_*i*_ ∈ *A* and *b*_*k*_ ∈ *C*, where *i*, *j*, and *k* take values from 1,…, *n*.

The values of *α*_*RoU*_(*t*_*i*_, *b*_*k*_) and *β*_*RoU*_(*t*_*i*_, *b*_*k*_) of the composition *T* = *R o U* are as follows (Table 1).

If the value of *T* is given by the following:

(48) $$\begin{array}{c} T=\alpha_{T}\left(t_{i},b_{k}\right)-\beta_{T}\left(t_{i},b_{k}\right)\cdot \pi_{T}\left(t_{i},b_{k}\right), \end{array}$$

then the student placement can be achieved.

### 5.1. Application Example

By using a hypothetical case with quasi-real data, we apply this method. Let *A*={*t*_1_, *t*_2_, *t*_3_, *t*_4_, *t*_5_} be the set of students for the colleges; *S* = {English Lang., Mathematics, Biology, Physics, Chemistry, Computer Sci.} be the set of related subject requirement to the set of colleges; and *C* = {College of Engineering (E), College of Medicine (M), College of Agricultural Engineering Sciences (AE), College of Sport Sciences (Sp), College of Science (S)} be the set of colleges the students are vying for (Algorithm 1).

From Table 4 and based on suitability of the students to the list of colleges, this decision making is made:

1. *t*_1_ and *t*_2_ are suitable to study at College of Agricultural Engineering Sciences.
2. *t*_3_ is suitable to study at College of Agricultural Engineering Sciences, College of Sport Sciences, and College of Science.
3. *t*_4_ is suitable to study at College of Medicine.
4. *t*_5_ is suitable to study at College of Agricultural Engineering Sciences and College of Science.

## 6. Discussion

**Figure 2:**
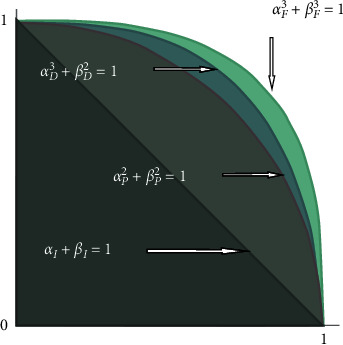
Comparison of grade space of IFSs, PFSs, FFSs, and (3, 2)-FSs.

The main idea of this work is to introduce a new type of fuzzy set called (3, 2)-FS. We illustrated that this type produces membership grades larger than intuitionistic and Pythagorean fuzzy sets which are already defined in the literature. However, Fermatean fuzzy sets give a larger space of membership grades than (3, 2)-FS.  Figure 2 illustrates the relationships between these types of fuzzy sets.


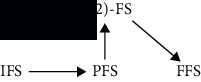


We summarize the relationships in terms of the space of membership and non-membership grades in the following figure.

Regarding topological structure, we illustrated that every fuzzy topology in the sense of Chang (intuitionistic fuzzy topology and Pythagorean fuzzy topology) is a (3, 2)-fuzzy topology. In contrast, every (3, 2)-fuzzy topological space is a Fermatean fuzzy topological space because every (3, 2)-fuzzy subset of a set can be considered as a Fermatean fuzzy subset. The next example elaborates that Fermatean fuzzy topological space need not be a (3, 2)-fuzzy topological space.

**Example 12 .**

Let *X*={*x*_1_, *x*_2_}. Consider the following family of Fermatean fuzzy subsets *τ*={1_*X*_, 0_*X*_, *D*_1_, *D*_2_}, where

(49) $$\begin{array}{c} D_{1}=\left\{\langle x_{1},\alpha_{D_{1}}\left(x_{1}\right)=0.75,\beta_{D_{1}}\left(x_{1}\right)=0.81\rangle ,\langle x_{2},\alpha_{D_{1}}\left(x_{2}\right)=0.85,\beta_{D_{1}}\left(x_{2}\right)=0.7\rangle \right\},\\ D_{2}=\left\{\langle x_{1},\alpha_{D_{2}}\left(x_{1}\right)=0.76,\beta_{D_{2}}\left(x_{1}\right)=0.81\rangle ,\langle x_{2},\alpha_{D_{2}}\left(x_{2}\right)=0.86,\beta_{D_{2}}\left(x_{2}\right)=0.7\rangle \right\}. \end{array}$$

Observe that (*X*, *τ*) is a Fermatean fuzzy topological space, but (*X*, *τ*) is not a (3, 2)-fuzzy topological space.

## 7. Conclusions

In this paper, we have introduced a new generalized intuitionistic fuzzy set called (3, 2)-fuzzy sets and studied their relationship with intuitionistic fuzzy, Pythagorean fuzzy, and Fermatean fuzzy sets. In addition, some operators on (3, 2)-fuzzy sets are defined and their relationships have been proved. The notions of (3, 2)-fuzzy topology, (3, 2)-fuzzy neighborhood, and (3, 2)-fuzzy continuous mapping were studied. Furthermore, we introduced the concept of (3, 2)-fuzzy points and studied separation axioms in (3, 2)-fuzzy topological space. We also introduced the concept of relation to (3, 2)-fuzzy sets, called (3, 2)-FR. Moreover, based on academic performance, the application of (3, 2)-FSs was explored on student placement using the proposed composition relation.

In future work, more applications of (3, 2)-fuzzy sets may be studied; also, (3, 2)-fuzzy soft sets may be studied. In addition, we will try to introduce the compactness and connectedness in (3, 2)-fuzzy topological spaces. The motivation and objectives of this extended work are given step by step in this paper.

## Figures and Tables

**Algorithm 1:**
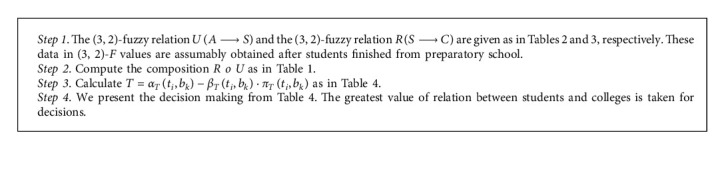
Determination of the optimal college for students.

**Table 1:** The composition *R* o *U*.

*R oU*	*E*	*M*	AE	Sp	*S*
*t*_1_	(0.81, 0.60)	(0.81, 0.60)	(0.81, 0.61)	(0.81, 0.60)	(0.81, 0.60)
*t*_2_	(0.82, 0.59)	(0.82, 0.60)	(0.82, 0.61)	(0.82, 0.59)	(0.82, 0.60)
*t*_3_	(0.82, 0.60)	(0.82, 0.60)	(0.82, 0.61)	(0.82, 0.61)	(0.82, 0.61)
*t*_4_	(0.82, 0.60)	(0.83, 0.60)	(0.82, 0.61)	(0.82, 0.61)	(0.82, 0.60)
*t*_5_	(0.83, 0.59)	(0.83, 0.59)	(0.83, 0.60)	(0.83, 0.59)	(0.83, 0.60)

**Table 2:** The (3, 2)-fuzzy relation *U*(*A*⟶*S*).

*U*(*A*⟶*S*)	Mathematics	Computer Sci.	English Lang.	Biology	Physics	Chemistry
*t*_1_	(0.81, 0.61)	(0.80, 0.62)	(0.81, 0.61)	(0.80, 0.61)	(0.71, 0.71)	(0.81, 0.60)
*t*_2_	(0.80, 0.61)	(0.81, 0.61)	(0.80, 0.61)	(0.62, 0.80)	(0.82, 0.60)	(0.82, 0.59)
*t*_3_	(0.82, 0.61)	(0.82, 0.60)	(0.82, 0.61)	(0.80, 0.62)	(0.62, 0.80)	(0.81, 0.61)
*t*_4_	(0.81, 0.62)	(0.83, 0.60)	(0.81, 0.61)	(0.81, 0.61)	(0.80, 0.61)	(0.82, 0.60)
*t*_5_	(0.83, 0.59)	(0.82, 0.60)	(0.83, 0.60)	(0.82, 0.59)	(0.81, 0.59)	(0.83, 0.59)

**Table 3:** The (3, 2)-fuzzy relation *R*(*S*⟶*C*).

*R*(*S*⟶*C*)	*E*	*M*	AE	Sp	*S*
Mathematics	(0.83, 0.59)	(0.84, 0.59)	(0.80, 0.62)	(0.82, 0.61)	(0.83, 0.60)
Computer Sci.	(0.82, 0.60)	(0.83, 0.59)	(0.80, 0.61)	(0.80, 0.62)	(0.80, 0.61)
English Lang.	(0.84, 0.59)	(0.83, 0.60)	(0.84, 0.59)	(0.83, 0.60)	(0.84, 0.59)
Biology	(0.81, 0.61)	(0.80, 0.609)	(0.80, 0.62)	(0.81, 0.61)	(0.81, 0.60)
Physics	(0.83, 0.60)	(0.82, 0.60)	(0.82, 0.61)	(0.82, 0.60)	(0.82, 0.60)
Chemistry	(0.83, 0.59)	(0.83, 0.60)	(0.82, 0.61)	(0.84, 0.59)	(0.83, 0.60)

**Table 4:** Greatest value given by *T*=*α*_*T*_(*t*_*i*_, *b*_*k*_) − *β*_*T*_(*t*_*i*_, *b*_*k*_) · *π*_*T*_(*t*_*i*_, *b*_*k*_).

*T*	*E*	*M*	AE	Sp	*S*
*t*_1_	0.425	0.425	0.434	0.425	0.425
*t*_2_	0.447	0.450	0.455	0.447	0.450
*t*_3_	0.450	0.450	0.455	0.455	0.455
*t*_4_	0.450	0.479	0.455	0.455	0.450
*t*_5_	0.474	0.474	0.479	0.474	0.479

## Data Availability

No data were used to support this study.
